# From congress to journal: publication outcomes of oral scientific presentations at the European society of urogenital radiology congresses

**DOI:** 10.3389/frma.2026.1897832

**Published:** 2026-07-29

**Authors:** Hamza Eren Güzel, Özgür Yaşar, Babak Saravi

**Affiliations:** 1 Department of Radiology, Izmir City Hospital, University of Health Sciences, Izmir, Türkiye; 2 Department of Oral, Maxillofacial and Facial Plastic Surgery, Medical Faculty and University Hospital Düsseldorf, Heinrich-Heine-University Düsseldorf, Düsseldorf, Germany

**Keywords:** bibliometrics, congress presentations, ESUR, journal quartile, publication rate, PubMed, scientific productivity, urogenital radiology

## Abstract

**Background/Objectives:**

Publication of congress presentations as full-text articles is an indirect indicator of scientific productivity and research quality. This study evaluated publication outcomes of oral scientific presentations delivered at European Society of Urogenital Radiology (ESUR) congresses and descriptively explored variation according to presentation characteristics.

**Methods:**

This retrospective bibliometric study included oral scientific presentations delivered at ESUR congresses between 2017 and 2022, excluding the canceled 2020 meeting. Poster presentations, invited lectures, educational sessions, and non-scientific sessions were excluded to maintain consistency with recent radiology congress publication studies. Presentations already published as full-text articles before the corresponding congress were excluded from the subsequent-publication denominator and reported separately. Publication status was determined by systematic PubMed/MEDLINE searches. The primary outcome was subsequent publication as a PubMed/MEDLINE-indexed full-text original research article by the final search date.

**Results:**

Among 183 oral presentations, 22 had been published before the corresponding congress and two were classified as ambiguous congress-month publications. After exclusion of pre-congress publications, 161 presentations were eligible for the main subsequent-publication analysis. During full available follow-up, 79 presentations were subsequently published, corresponding to a publication proportion of 49.1% (95% CI, 41.1–57.0%). The median time to publication was 12.9 months, and 69 of 79 subsequent publications (87.3%) appeared within 36 months.

**Conclusions:**

ESUR oral scientific presentations showed a subsequent publication proportion comparable to contemporary European radiology congresses. The use of oral-only inclusion, exclusion of pre-congress publications, and PubMed/MEDLINE-based publication matching aligns this study with recent ECR, ESGAR, and ESNR publication-outcome analyses.

## 1 Introduction

Scientific congresses play a central role in the dissemination of original research, academic exchange, and the development of scientific collaboration. Oral scientific presentations often represent mature or high-priority research selected for formal discussion. Although congress abstracts allow early communication of findings, only a proportion subsequently progress to full-text publication in peer-reviewed journals. Therefore, the publication rate of congress presentations is widely used as an indirect indicator of the scientific productivity, research quality, and academic output of a meeting (Scherer et al., 2018; Grover et al., 2021).

The conversion of congress presentations into full-text publications has been evaluated across several medical specialties, with reported rates varying substantially according to specialty, meeting type, follow-up duration, database selection, and search strategy (Scherer et al., 2018; Grover et al., 2021). Recent non-radiology congress-publication studies have also emphasized the relevance of pre-congress publication status, presentation format, study characteristics, and funding in understanding abstract-to-publication patterns (Weber et al., 2026; Oetzmann von Sochaczewski et al., 2023). In radiology, earlier studies evaluating the European Congress of Radiology (ECR) demonstrated publication rates of approximately 45%−47% for presentations delivered in 2000 and 2001, while subsequent ECR analyses reported rates of approximately 43%−52% depending on methodological choices (Miguel-Dasit et al., 2006a,b; Secil et al., 2007; Loughborough et al., 2016; Dollinger et al., 2018).

More recent radiology-focused studies have further emphasized the importance of standardized methodology. An ECR 2019 analysis applied a uniform follow-up period to enable comparison with ECR 2010 and analyzed publication outcomes according to country, collaboration type, modality, subspecialty, study design, journal impact, and citation metrics (Salbas et al., 2026). Recent ESGAR and ESNR analyses included congresses up to 2022 because data collection in 2025 allowed at least approximately 3 years of follow-up, and they also examined country of origin, collaboration patterns, study design, time to publication, journal impact, and citation metrics (Salbas and Kaya, 2026; Koc and Salbas, 2026).

Recent publication-outcome studies have updated contemporary data for major European radiology congresses, including ECR, ESGAR, and ESNR, using broadly similar methodology based on oral scientific presentations, exclusion of pre-congress publications, and PubMed/MEDLINE-indexed full-text original research articles (Salbas et al., 2026; Salbas and Kaya, 2026; Koc and Salbas, 2026). However, the publication outcomes of ESUR oral scientific presentations have not previously been systematically evaluated. The present study extends this methodological framework to urogenital radiology by evaluating the subsequent publication proportion, time to publication, and journal characteristics of ESUR oral scientific presentations. To maintain comparability with recent radiology congress studies, the primary outcome was subsequent PubMed/MEDLINE-indexed full-text original research publication by the final search date, and time to publication was analyzed descriptively.

## 2 Materials and methods

### 2.1 Study design and data source

This retrospective bibliometric study evaluated the publication outcomes of oral scientific presentations delivered at the annual ESUR congresses between 2017 and 2022. The 2020 ESUR congress was not included because the meeting was canceled during the COVID-19 pandemic. The 2021 ESUR congress was held online, which may have contributed to the lower number of oral presentations in that year.

The list of oral scientific presentations was obtained from the official ESUR congress website and the publicly available oral presentation lists/programs for each included congress year. For each year, eligible oral scientific presentations were identified from the official ESUR oral scientific presentation sections. The extracted presentation list used for the analysis is provided as Supplementary Table S1.

Only oral scientific presentations were included. Poster presentations were excluded to maintain methodological consistency with recent radiology congress publication-outcome studies, including ECR, ESGAR, and ESNR, which focused on oral presentations, and to avoid heterogeneity introduced by combining presentation formats with different selection processes and expected publication probabilities. Educational lectures, invited talks, workshops, poster presentations, non-scientific sessions, and duplicate entries were excluded. Presentations that had already been published as full-text articles before the first day of the corresponding ESUR congress were identified separately and excluded from the main subsequent-publication denominator.

As this study used publicly available congress and publication data and did not involve patients, patient-level data, or human participants, institutional review board approval and informed consent were not required.

### 2.2 Data extraction and variable definitions

For each oral presentation, the following variables were extracted: congress year, abstract title, first author, country of origin, urogenital subspecialty category, imaging modality, artificial intelligence-related content, publication status, publication title, journal name, publication date, journal quartile, and time from congress presentation to publication. Journal quartiles were determined using Journal Citation Reports according to the Science Citation Index Expanded category of each journal. When possible, the quartile corresponding to the year of publication was used; if unavailable, the closest available year was used. Journals not indexed in SCIE were classified separately as ESCI or N/A, as applicable.

The country of origin was defined according to the affiliation or location of the first author as reported in the congress materials. Country names were standardized before analysis. Urogenital subspecialty categories were standardized as prostate, upper urinary tract, lower urinary tract, male reproductive system, female reproductive system, and other. Imaging modality was categorized as MRI, CT, ultrasound, PET, radiography, nuclear medicine, multimodality, other, or unspecified. Presentations involving more than one imaging modality were classified as multimodality.

Presentations were classified as artificial intelligence-related if the title or available abstract information clearly indicated the use of artificial intelligence, machine learning, deep learning, radiomics, or related computational methods. Study design was not analyzed because the available ESUR congress materials did not consistently provide sufficient methodological detail to reliably classify presentations as retrospective, prospective, or other. This approach was chosen to avoid misclassification bias.

Study design, funding source, and collaboration type were not included as main predictor variables. Although these variables have been evaluated in recent ECR, ESGAR, and ESNR analyses, the available ESUR congress materials did not consistently provide sufficient abstract-level methodological detail or complete affiliation-level information for unpublished presentations. Classifying these variables from titles alone was considered unreliable and at high risk of misclassification. Therefore, these variables were not analyzed as predictors in the main study.

### 2.3 Publication search strategy and matching

Publication status was determined by a systematic PubMed/MEDLINE search using a predefined strategy adapted from recent ECR, ESGAR, and ESNR publication outcome studies (Salbas et al., 2026; Salbas and Kaya, 2026; Koc and Salbas, 2026). Searches initially used the surname and initials of the first author. If no corresponding publication was identified, searches were repeated using co-author names and key terms from the abstract title. When multiple potential matches were retrieved, the title, authorship, study topic, methodology, and target population were compared with the original congress presentation.

PubMed/MEDLINE was selected as the primary publication database for three reasons. First, it provides a reproducible and conservative method for identifying peer-reviewed biomedical publications. Second, it allowed methodological comparability with recent ECR, ESGAR, and ESNR publication-outcome studies, which also used PubMed/MEDLINE-based searches. Third, restricting the primary outcome to PubMed/MEDLINE-indexed full-text original research articles reduced heterogeneity related to database coverage and article type. Publications indexed only in other databases were not included in the primary outcome; therefore, the reported publication proportion should be interpreted as a conservative PubMed/MEDLINE-indexed publication proportion rather than the total number of all possible peer-reviewed publications.

Publication status was independently checked by two investigators using the predefined PubMed/MEDLINE search strategy and matching criteria. Interobserver agreement was calculated for the initial publication-status classification before consensus review. Discrepant or uncertain cases were resolved by joint re-evaluation and consensus.

Reviews, editorials, pictorial essays, case reports, letters/correspondence items, and non-indexed articles were excluded from the primary outcome. This restriction was used to maintain consistency with recent radiology congress publication studies and to define a conservative outcome limited to full-text original research articles.

The final publication search was completed in January 2026, ensuring a minimum potential follow-up period of at least 36 months for all included congress years, including the most recent eligible congress year, ESUR 2022.

### 2.4 Publication timing and outcome measures

The publication date was recorded as the earliest available online publication date when available. If an online-first date was not available, the issue publication date was used. Time to publication was calculated as the interval between the first day of the corresponding ESUR congress and the publication date, expressed in months.

Presentations were classified into three main publication-status groups: subsequently published, previously published before congress, and unpublished. Presentations with month-only publication dates overlapping with the congress month were classified as ambiguous congress-month publications rather than being forced into pre-congress or subsequent publication status.

The primary outcome was the subsequent publication proportion, defined as the number of eligible oral presentations published as PubMed/MEDLINE-indexed full-text original research articles by the final search date divided by the number of eligible oral presentations after exclusion of pre-congress publications.

Time to publication was analyzed descriptively using predefined intervals of 0–12, 13–24, 25–36, 37–48, 49–60, and >60 months. The proportion of subsequent publications appearing within the first 36 months was reported descriptively because previous radiology congress studies have shown that most abstracts that eventually reach publication do so within approximately 3 years after presentation.

Secondary outcomes included publication proportion by congress year, urogenital subspecialty, imaging modality, country/region of origin, AI-related content, and journal quartile distribution.

### 2.5 Statistical analysis

Descriptive statistics were used to summarize the characteristics of oral presentations and subsequent publications. Categorical variables were reported as frequencies and percentages. Continuous variables were reported as mean ± standard deviation or median with interquartile range, depending on data distribution. Normality of continuous variables was assessed using the Shapiro–Wilk test and visual inspection of histograms and Q-Q plots.

Publication proportions were reported with 95% confidence intervals. Although the term “publication rate” is commonly used in the congress-publication literature, the outcome in this study statistically represents a publication proportion within a defined follow-up period. Interobserver agreement for publication-status classification was assessed using Cohen's κ.

Exploratory comparisons of publication proportions across congress year, subspecialty category, imaging modality, country/region group, and AI-related status were performed using chi-square tests or Fisher's exact tests, as appropriate. Fisher's exact test was used when expected cell counts were small. Because of the limited sample size and sparse events in several subgroups, no multivariable logistic regression was performed. Country-level analyses were considered descriptive/exploratory and were not interpreted as evidence of independent country effects.

No adjustment for multiple comparisons was performed; therefore, subgroup *p*-values were interpreted as nominal and hypothesis-generating. A two-sided *p*-value of less than 0.05 was considered statistically significant for exploratory analyses.

## 3 Results

### 3.1 Overall publication outcomes

A total of 183 oral scientific presentations delivered at ESUR congresses between 2017 and 2022 were identified. Among all presentations, 22 had already been published before the start date of the corresponding ESUR congress and were excluded from the subsequent-publication denominator. Two additional presentations had month-only publication dates overlapping with the congress month and were classified as ambiguous congress-month publications rather than being forced into pre-congress or subsequent publication status.

After exclusion of pre-congress publications, 161 presentations remained eligible for the main subsequent-publication analysis. During full available follow-up, 79 eligible presentations were subsequently published as PubMed/MEDLINE-indexed full-text original research articles, corresponding to a publication proportion of 49.1% (95% CI, 41.1–57.0%). When both pre-congress and ambiguous congress-month publications were excluded from the denominator, the strict publication proportion was 49.7%. Interobserver agreement for publication-status classification before consensus review was 98.9% (181/183), with a Cohen's κ of 0.982, indicating almost perfect agreement. The two discrepant cases were resolved by joint consensus review. Overall publication outcomes are summarized in Table 1.

**Table 1:** Overall publication outcomes.

Metric	Value
Total oral presentations	183
Pre-congress publications	22
Ambiguous congress-month publications	2
Eligible presentations after exclusion of pre-congress publications	161
Subsequent publications during full available follow-up	79
Primary publication proportion during full available follow-up	49.1%
95% CI for primary publication proportion	41.1–57.0%
Strict publication proportion excluding ambiguous congress-month publications from denominator	49.7%
Subsequent publications appearing within 36 months	69/79 (87.3%)
Unpublished during full available follow-up	80

The primary denominator excluded pre-congress publications but retained ambiguous congress-month publications within the eligible cohort. The strict publication proportion excluded both pre-congress and ambiguous congress-month publications from the denominator.

**Figure 1:**
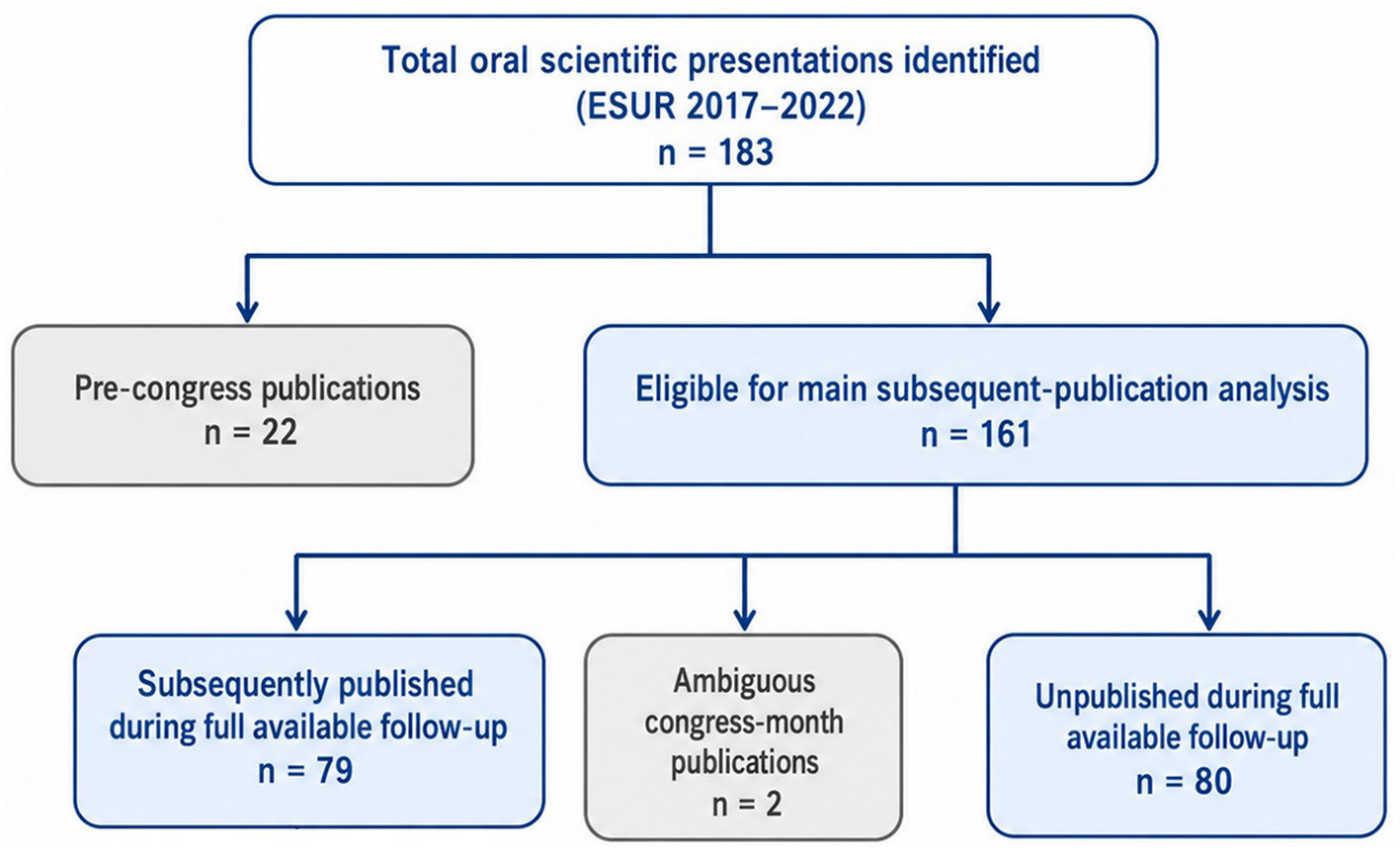
Study selection and publication-status flow of ESUR oral presentations. Pre-congress publications were excluded from the denominator of the main subsequent-publication analysis. Ambiguous congress-month publications were retained within the eligible cohort but were classified separately and were not forced into pre-congress or subsequent-publication categories.

Pre-congress publications accounted for 12.0% of all identified oral presentations. These presentations were reported separately because they did not represent post-congress publication events. Overall study selection and publication-status flow are summarized in Figure 1.

### 3.2 Publication outcomes by congress year

**Figure 2:**
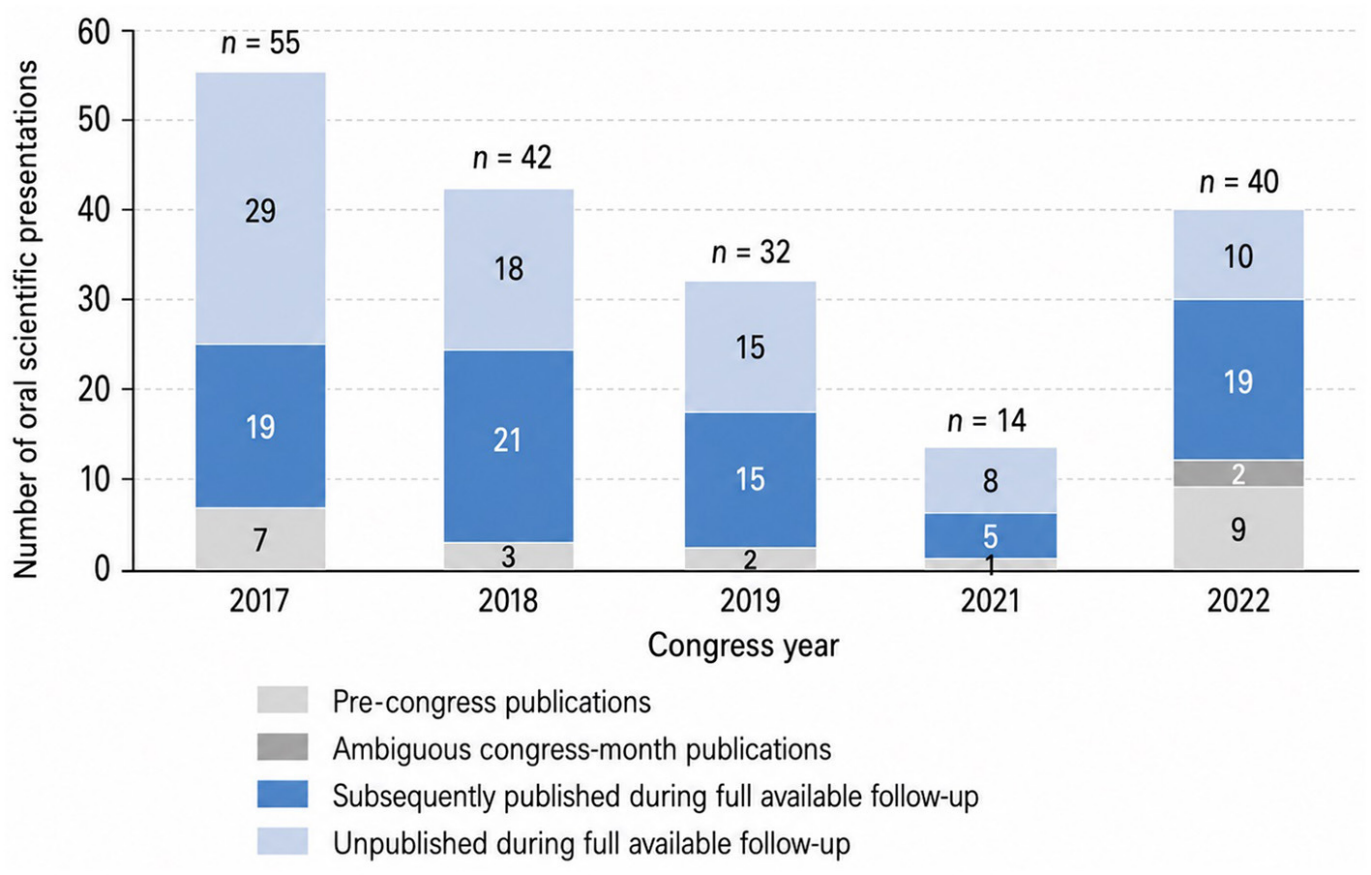
Publication outcomes of ESUR oral scientific presentations by congress year. Each stacked bar represents all oral scientific presentations identified in that year and is subdivided into pre-congress publications, ambiguous congress-month publications, subsequently published presentations during full available follow-up, and unpublished presentations.

The number of eligible oral presentations varied across congress years. During full available follow-up, the highest publication proportion was observed for ESUR 2022 (61.3%), followed by 2018 (53.8%) and 2019 (50.0%). The publication proportions were 39.6% for 2017 and 38.5% for 2021. No statistically significant difference in publication proportion was observed across congress years (*p* = 0.339). Publication outcomes by congress year are shown in Figure 2, and detailed values are provided in Supplementary Table S2.

### 3.3 Time to publication

**Figure 3:**
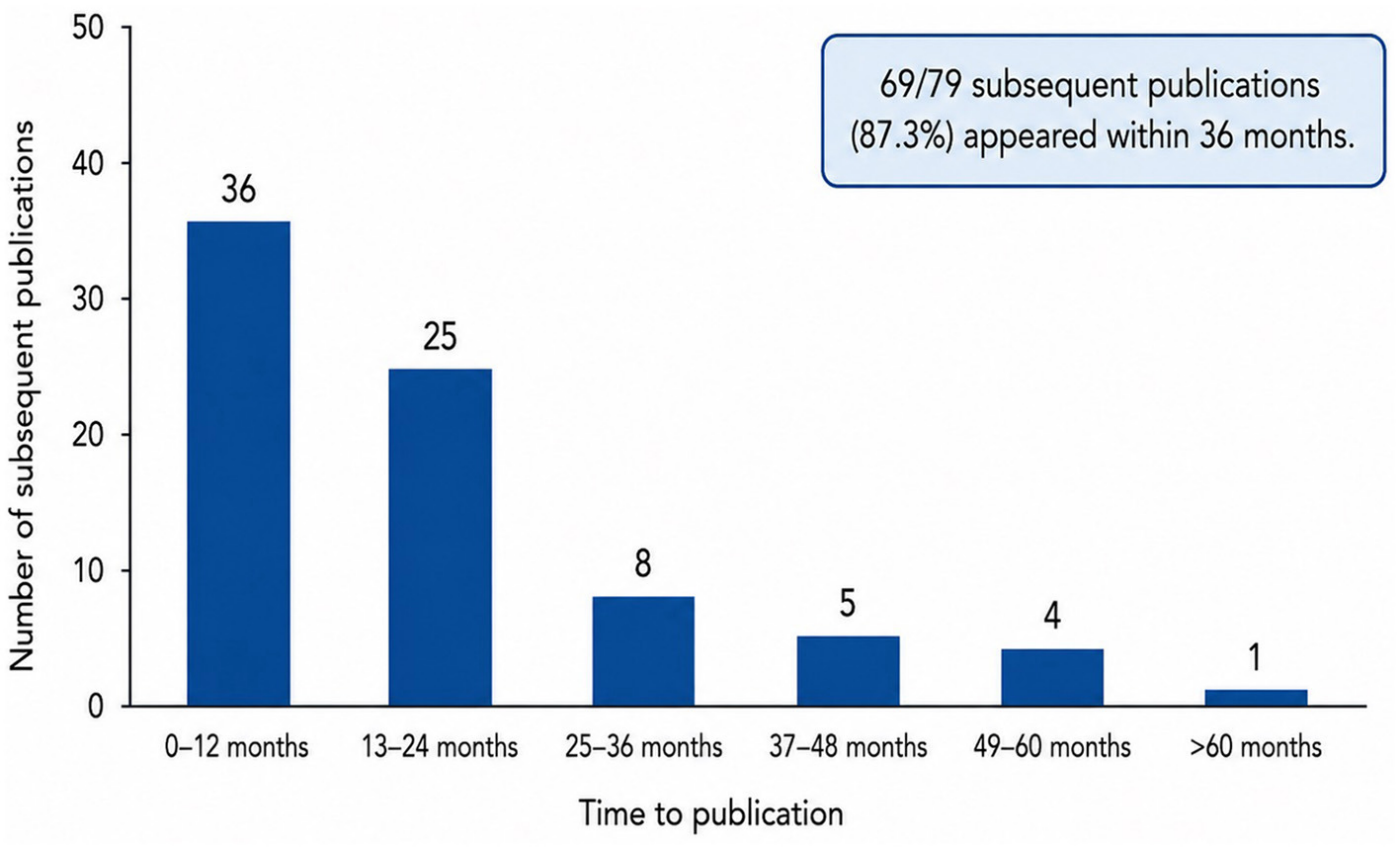
Time from ESUR oral presentation to subsequent full-text publication. Bars show the number of subsequent publications identified during full available follow-up according to time interval from congress presentation to publication.

Among the 79 subsequent publications identified during full available follow-up, the median time from congress presentation to publication was 12.9 months (interquartile range, 6.2–23.8 months; range, 0.1–89.8 months). Thirty-six publications (45.6%) appeared within 12 months, 61 (77.2%) within 24 months, and 69 (87.3%) within 36 months after presentation. The distribution of time to publication is shown in Table 2 and Figure 3.

**Table 2:** Time from ESUR congress presentation to publication.

Publication time	*n*	%	Cumulative %
0–12 months	36	45.6%	45.6%
13–24 months	25	31.6%	77.2%
25–36 months	8	10.1%	87.3%
37–48 months	5	6.3%	93.7%
49–60 months	4	5.1%	98.7%
>60 months	1	1.3%	100.0%
Total	79	100.0%	-

Percentages were calculated among the 79 subsequent publications identified during full available follow-up.

### 3.4 Publication outcomes by urogenital subspecialty

Publication proportions varied across urogenital subspecialty categories. The highest publication proportion was observed in male reproductive imaging (15/20, 75.0%), followed by upper urinary tract imaging (24/42, 57.1%) and female reproductive imaging (18/33, 54.5%). Lower urinary tract imaging (4/16, 25.0%) and other topics (1/7, 14.3%) showed lower publication proportions. This subgroup comparison was exploratory because several categories contained small numbers of presentations. Detailed exploratory results by urogenital subspecialty are provided in Supplementary Table S3.

### 3.5 Publication outcomes by imaging modality

MRI was the most common imaging modality among eligible presentations. During full available follow-up, publication proportions were 57.4% for MRI, 54.3% for CT, 44.4% for ultrasound, 62.5% for multimodality studies, and 12.5% for unspecified or non-modality-specific presentations. Publication proportions appeared to vary across modality categories, mainly because of the low publication proportion in unspecified or non-modality-specific presentations. After exclusion of unspecified/non-modality-specific presentations, no clear modality-related pattern was evident. Because several modality categories contained very small numbers of presentations, these findings were considered exploratory.

### 3.6 Publication outcomes by country of origin

Country-level analyses were performed descriptively because of the limited number of eligible presentations in several country groups. During full available follow-up, Italy had the highest publication proportion among countries analyzed separately (20/27, 74.1%). Publication proportions were 50.0% for South Korea, 42.9% for Sweden, 23.5% for the United Kingdom, and 47.2% for the grouped other countries category. Because country assignment was based on first-author affiliation and several groups were small, these findings were interpreted as descriptive rather than evidence of independent country-level effects. Detailed descriptive country-group results are provided in Supplementary Table S5.

### 3.7 Artificial intelligence-related presentations

Eleven presentations were classified as AI-related. After exclusion of two pre-congress publications, nine AI-related presentations remained eligible for analysis. Because of the small number of AI-related presentations, this subgroup was analyzed descriptively and interpreted as hypothesis-generating. Detailed descriptive results for AI-related and non-AI presentations are provided in Supplementary Table S6.

### 3.8 Journal quartile distribution

Among the 79 subsequent publications identified during full available follow-up, 51 articles (64.6%) were published in SCIE Q1 journals and 9 articles (11.4%) in SCIE Q2 journals. Overall, 60 of 79 subsequent publications (75.9%) appeared in SCIE Q1 or Q2 journals. Fifteen publications (19.0%) appeared in SCIE Q3 journals, one (1.3%) in a SCIE Q4 journal, one (1.3%) in an ESCI Q3 journal, and two publications (2.5%) were classified as N/A. The journal quartile distribution is shown in Table 3.

**Table 3:** Journal quartile distribution of subsequent publications during full available follow-up.

Journal category	*n*	%
SCIE Q1	51	64.6%
SCIE Q2	9	11.4%
SCIE Q3	15	19.0%
SCIE Q4	1	1.3%
ESCI Q3	1	1.3%
N/A	2	2.5%
Total	79	100.0%

## 4 Discussion

In this retrospective bibliometric study, approximately half of the eligible oral scientific presentations delivered at ESUR congresses between 2017 and 2022 were subsequently published as PubMed/MEDLINE-indexed full-text original research articles. The overall publication proportion was 49.1%, with a median time to publication of 12.9 months. Most subsequent publications appeared within the first 3 years after presentation, and three quarters of the published articles appeared in SCIE Q1 or Q2 journals.

The publication proportion observed in the present ESUR cohort is closely aligned with recent European radiology congress data. In the ECR 2019 analysis, 46.5% of oral presentations were subsequently published, while the corresponding proportions were 52.8% for ESGAR 2019–2022 and 47.3% for ESNR 2018–2022 (Salbas et al., 2026; Salbas and Kaya, 2026; Koc and Salbas, 2026). A summary comparison with contemporary European radiology congress publication studies is provided in Table 4. Therefore, although ESUR is a smaller and more subspecialized congress, its publication outcome appears broadly comparable to both large general radiology meetings and other European subspecialty radiology congresses. This supports the interpretation that oral scientific presentations at ESUR represent a research output with publication potential similar to that of other major European radiology meetings.

**Table 4:** Comparison with contemporary European radiology congress publication studies.

Congress	Congress years	Eligible presentations	Published articles	Publication proportion	Follow-up approach
ECR	2019	1,817	844	46.5%	Uniform 4 years 9 months
ESGAR	2019–2022	407	215	52.8%	Meetings up to 2022; data collection in 2025
ESNR	2018–2022	241	114	47.3%	Meetings up to 2022; data collection in 2025
Present ESUR study	2017–2022	161	79	49.1%	Meetings up to 2022; data collection in 2026

Our findings also compare favorably with earlier radiology congress studies. Arrivé et al. (2004) reported that 33.6% of orally presented original studies from the 1995 RSNA Scientific Assembly were subsequently published within 5 years. Marx et al. (1999) found a similar publication proportion of 37% for neuroradiologic abstracts presented at national meetings in 1993. Publication proportions reported from national radiology congresses have generally been lower; for example, Secil et al. (2005) reported publication proportions of 15.4% for oral presentations and 11.1% for poster presentations at Turkish national radiology congresses. Ha et al. (2008) also reported a lower overall publication proportion of 27.4% for abstracts presented by Korean investigators at major radiology meetings, although publication proportions varied according to the meeting type. Against this background, the 49.1% publication proportion observed for ESUR is in the upper range of historical radiology congress outcomes.

More recent radiology subspecialty studies have reported publication proportions closer to the present findings. Dangouloff-Ros et al. (2015a) reported that 40% of orally presented abstracts from the French National Congress of Radiology were subsequently published. In the focused analysis of French abstracts from the same congress, the publication proportion was 43%, again close to the present ESUR result (Dangouloff-Ros et al., 2015b). Shergill et al. (2017) reported an overall publication proportion of 44.9% for abstracts presented at major interventional radiology conferences. Tritz et al. (2018) found that 50.6% of Skeletal Society of Radiology annual meeting abstracts were subsequently converted to full-text publications. These results suggest that publication proportions around 45–55% are typical for selected research presentations at established radiology and radiology-subspecialty meetings, and the ESUR result fits well within this range.

The time-to-publication profile in the present study was also consistent with prior literature. The median time to publication was 12.9 months, and 87.3% of subsequent publications appeared within 36 months. Ha et al. (2008) reported a mean time to publication of 15.8 months for abstracts presented by Korean investigators at major radiology meetings. Shergill et al. (2017) reported a mean time to publication of approximately 15 months for interventional radiology conference abstracts. Tritz et al. (2018) reported a median time to publication of 13 months for Skeletal Society of Radiology abstracts, closely matching the present ESUR finding. The observation that most ESUR publications appeared within 3 years is also consistent with recent ECR, ESGAR, and ESNR analyses, supporting the adequacy of the available follow-up period without restricting the primary outcome to a fixed 36-month cutoff (Salbas et al., 2026; Salbas and Kaya, 2026; Koc and Salbas, 2026).

The journal profile of subsequent ESUR publications suggests that many studies reached relatively high-ranking journals. Among the 79 subsequent publications, 64.6% appeared in SCIE Q1 journals and 75.9% appeared in SCIE Q1 or Q2 journals. Although journal quartile is an imperfect surrogate for scientific impact, this distribution indicates that ESUR oral presentations not only convert to full-text articles at a proportion comparable to other European radiology meetings, but also frequently reach higher-quartile journals. Previous radiology congress studies have similarly evaluated publication venue, journal impact, and citation-related outcomes as markers of the academic visibility of congress presentations (Salbas et al., 2026; Salbas and Kaya, 2026; Koc and Salbas, 2026; Dangouloff-Ros et al., 2015a; Tritz et al., 2018).

The exclusive use of PubMed/MEDLINE also has implications beyond simple database coverage. MEDLINE indexing is selective and predominantly captures biomedical journals with international visibility, many of which publish in English. Therefore, a PubMed/MEDLINE-only strategy may undercount full-text publications appearing in national or regional journals, particularly when researchers from non-English-speaking countries publish in their mother tongue. This is especially relevant in Europe, where viable scientific publication landscapes exist in major languages such as French, Spanish, German, Italian, Portuguese, and Dutch. Consequently, the present approach may differentially underestimate dissemination by non-English-speaking authors and should not be interpreted as capturing the complete publication output of all ESUR presentations. Similar concerns regarding language- and database-related visibility have been discussed in congress-publication research (Oetzmann von Sochaczewski et al., 2023). For this reason, our estimate should be interpreted specifically as the proportion of ESUR oral presentations converted into PubMed/MEDLINE-indexed full-text original research articles.

The restriction to oral presentations is another important consideration. Presentation format is not a neutral variable in abstract-to-publication studies. Oral presentations are usually more selectively chosen, may represent more mature or higher-priority research, and are often more likely to progress to full-text publication than poster presentations. For example, previous combined-format studies have reported separate publication proportions for oral and poster presentations and have shown that presentation format may influence subsequent publication, although this association is not uniform across all cohorts (Grover et al., 2021; Oetzmann von Sochaczewski et al., 2023; Secil et al., 2005; Ha et al., 2008). The present study was intentionally designed as an oral-presentation analysis to align with recent ECR, ESGAR, and ESNR studies; however, this design means that the results cannot be generalized to the entire ESUR congress output. The overall publication proportion of all ESUR presentations would likely differ if poster presentations were included.

Pre-congress publications also require specific interpretation. These studies differ conceptually from subsequent publications because the full-text article already existed before the congress presentation. Including them in the subsequent-publication denominator would therefore overestimate post-congress conversion from abstract to article. At the same time, excluding them without separate reporting would hide a relevant feature of congress dissemination. In the present study, 12.0% of all ESUR oral presentations had already been published before the corresponding congress, indicating that ESUR congresses serve not only as a platform for preliminary research but also as a venue for disseminating recently published work to a focused subspecialty audience. The proportion of pre-congress publications may be influenced by abstract submission deadlines, online-first publication practices, journal processing times, and congress policies regarding already published material. Therefore, pre-congress publications should be interpreted as a separate dissemination category rather than as either unpublished abstracts or post-congress publication events. This approach avoids inflation of the subsequent-publication proportion while preserving information about already published research presented at the congress (Oetzmann von Sochaczewski et al., 2023; Salbas et al., 2026; Salbas and Kaya, 2026; Koc and Salbas, 2026).

Exploratory subgroup analyses provided descriptive insight into the ESUR research landscape. Publication proportions appeared higher for male reproductive imaging, upper urinary tract imaging, and female reproductive imaging, whereas lower urinary tract imaging and other topics showed lower proportions. These differences may partly reflect variation in sample size, maturity of research questions, availability of standardized imaging pathways, and the likelihood that certain topics are more frequently developed into full manuscripts. Similar subspecialty-related variation has been reported in previous radiology congress studies, including the ECR 2019 analysis and the French National Congress of Radiology analyses (Salbas et al., 2026; Dangouloff-Ros et al., 2015a,b). However, because several ESUR subspecialty categories contained small numbers of presentations, these findings should be interpreted as descriptive patterns rather than independent predictors of publication.

Imaging modality and country-level findings should be interpreted with similar caution. MRI and CT accounted for most eligible ESUR presentations and showed publication proportions above the overall cohort average, whereas unspecified or non-modality-specific presentations had a lower publication proportion. This may suggest that clearly defined imaging-based studies are more likely to progress to full-text publication than broader or less modality-specific presentations, but this interpretation remains exploratory. Country-level differences were also descriptive only, because country assignment was based on first-author affiliation and several country groups were small. The AI-related subgroup showed a numerically high publication proportion, consistent with the strong academic visibility of AI/ML research reported in the ECR 2019 analysis (Salbas et al., 2026), but only nine AI-related presentations remained eligible after exclusion of pre-congress publications. Therefore, AI-related findings should be regarded as hypothesis-generating.

This study has several limitations. First, publication searches were restricted to PubMed/MEDLINE-indexed full-text original research articles. Although this provided a reproducible and conservative benchmark comparable with recent radiology congress studies, it may have underestimated the true publication proportion. In particular, the PubMed/MEDLINE-based strategy may have missed articles published in non-indexed journals, journals indexed only in Scopus, Web of Science, Embase, Google Scholar, or other databases, and national or regional non-English journals. This limitation may be especially relevant for non-English-speaking authors who publish full-text work in their mother tongue. Second, only oral scientific presentations were included. Because oral presentations are generally more selectively chosen than posters, the present findings should be interpreted as publication outcomes of a selected subset of ESUR congress research and cannot be generalized to the entire congress output. Third, study design, funding source, and collaboration type could not be reliably analyzed because the available ESUR congress materials did not consistently provide sufficient methodological or affiliation-level detail for unpublished presentations. Fourth, publication matching was based on author names, title similarity, study topic, methodology, and target population; although ambiguous cases were reviewed by multiple investigators, some matches may have been missed if titles or author lists changed substantially. Fifth, the classification of pre-congress publications depended on available online-first or issue publication dates. Although ambiguous congress-month publications were classified separately to avoid forcing uncertain cases into either pre-congress or subsequent-publication categories, publication-date granularity may still have affected the classification of a small number of borderline cases. Finally, subgroup analyses were exploratory, involved small numbers in several categories, and were not adjusted for multiple comparisons.

## 5 Conclusions

In conclusion, ESUR oral scientific presentations showed a subsequent PubMed/MEDLINE-indexed publication proportion comparable to contemporary ECR, ESGAR, and ESNR data and generally within the upper range of historical radiology congress publication outcomes. Most subsequent publications appeared within 3 years, and a substantial majority were published in SCIE Q1 or Q2 journals. These findings extend the current European radiology congress publication-outcome literature to urogenital radiology and suggest that ESUR oral scientific presentations have publication outcomes broadly consistent with other major European radiology and subspecialty congresses.

## Data Availability

The original contributions presented in the study are included in the article/Supplementary material, further inquiries can be directed to the corresponding author.
