# Supplementary material for: From congress to journal: publication outcomes of oral scientific presentations at the European society of urogenital radiology congresses
Source: Front Res Metr Anal. 2026 Jul 29;11:1897832. doi: 10.3389/frma.2026.1897832 (PMC13461717; doi:10.3389/frma.2026.1897832)
Supplement: Supplementary file 1 [file Table_1.docx]

**Table S1. Oral scientific presentations delivered at ESUR congresses included in the analysis**

*ESUR congress years: 2017, 2018, 2019, 2021, and 2022. The 2020 congress was not included because the meeting was cancelled during the COVID-19 pandemic.*

| **No.** | **Congress year** | **Presentation title** | **Author(s)** |
| --- | --- | --- | --- |
| 1 | 2017 | Role of multi-parametric MRI of the prostate to detect significant prostate cancer | Victoria Josefa Gomez Martinez, Silvan Boxler, Harriet Thoeny, Sebastiano Barbieri |
| 2 | 2017 | Prostate carcinoma: does the addition of PSMA-PET change staging? | Jane Belfield, Priya Healey, Sobhan Vinjamuri |
| 3 | 2017 | PSMA — The experience so far | Jane Belfield, Priya Healey, Maggie Cooper, Sobhan Vinjamuri |
| 4 | 2017 | Estimation of the highest gleason score with multiparametric magnetic resonance imaging — transrectal ultrasound guided fusion biopsy compared to standard systematic biopsy. Has the time come to rely on targeted biopsy? | Giovanni Barchetti, Maurizio Del Monte, Antonella Santodirocco, Martina Pecoraro, Isabella Ceravolo, Valeria Panebianco |
| 5 | 2017 | Improvement of prostate cancer detection rate using TRUS-MRI targeted biopsy: the role of MpMRI associated to Watson Elementary CAD system | Riccardo Campa, Maurizio Del Monte, Vincenzo Salvo, Martina Pecoraro, Carlo Catalano, Valeria Panebianco |
| 6 | 2017 | Multiparametric MRI for detection of extracapsular extension in prostate cancer: use of a 1.5 T MRI system with a torso coil | Ahmet Baki Yagci, Ergin Sagtas, Zafer Aybek, Nilay Sen Turk, Duygu Herek, Ali Ersin Zumrutbas |
| 7 | 2017 | PI-RADS v2 for detection of prostate cancer: use of a 1.5 T MRI system with torso coil | Ahmet Baki Yagci, Ergin Sagtas, Omer Levent Tuncay, Nilay Sen Turk, Pinar Cakmak, Tahir Turan |
| 8 | 2017 | Prospective study of diagnostic accuracy comparing prostate cancer detection by transrectal ultrasound biopsy versus MR/US fusion biopsy | Filipp Kossov, Vladimir Kapustin, Bakhodur Kamolov, Iskander Abdulllin, Maksim Shorikov, Vadim Panov |
| 9 | 2017 | The value of whole-lesion apparent diffusion coefficient. Analysis for predicting progression of prostate cancer active surveillance | Tsutomu Tamada, Hasan Dani, Samir S. Taneja, Andrew B. Rosenkrantz |
| 10 | 2017 | Color-doppler ultrasound, clinical and seminal correlations of prostatitis-like symptoms in males of infertile couples | Francesco Lotti, Giovanni Corona, Nicola Mondaini, Elisa Maseroli, Matteo Rossi, Erminio Filimberti, Ivo Noci, Gianni Forti, Mario Maggi |
| 11 | 2017 | Clinical and male genital tract ultrasound correlates of premature ejaculation in men with couple infertility | Francesco Lotti, Giovanni Corona, Giulia Rastrelli, Gianni Forti, Emmanuele Jannini, Mario Maggi |
| 12 | 2017 | Seminal vesicles ultrasound features in a cohort of infertility patients | Francesco Lotti, Giovanni Corona, Giovanni Maria Colpi, Erminio Filimberti, Selene Degl'Innocenti, Mario Mancini, Elisabetta Baldi, Ivo Noci, Gianni Forti, Mario Maggi |
| 13 | 2017 | Role of contrast-enhanced sonography in diagnosis of paediatric scrotal pathology | Dean Huang, Rashmeet Chhabra, Maria Sellars, Annamaria Deganello, Paul Sidhu |
| 14 | 2017 | Contrast-enhanced ultrasound in the differential diagnosis of small testicular masses | Beniamino Corcioni, Caterina Gaudiano, Alessandro Franceschelli, Giorgio Gentile, Riccardo Schiavina, Rita Golfieri |
| 15 | 2017 | Multiple, synchronous nodules of different histology within the same testis: ultrasonographic/pathologic correlations | Lorenzo E. Derchi, Bertolotto M, Cicero C, Hawthorn B, Trambaiolo Antonelli C, Sidhu P, Ascenti V, Nikolaidis P, Dudea S, Toncini C |
| 16 | 2017 | Magnetic resonance diffusion tensor imaging of normal and pathologic testis: preliminary observations | Athina Tsili, Alexandra Ntorkou, Loukas Astrakas, Ekaterini Boukali, Dimitrios Giannakis, Vasilios Maliakas, Nikolaos Sofikitis, Maria I. Argyropoulou |
| 17 | 2017 | Lithiasis-induced acute kidney injury (AKI) | Jeries Paolo Zawaideh, Michele Bertolotto, Lorenzo Egildo Derchi |
| 18 | 2017 | CT guided cryoablation of small renal masses | Janni L.B. Madsen, Allan L. Pedersen, Jonas A. Bojsen, Ole Graumann |
| 19 | 2017 | Post-operative complications following gender reassignment surgery | Jane Belfield, Ammad Farooq |
| 20 | 2017 | Quantitative analysis of cervical cancer extent to parametrium using different MRI modalities | Elena Tarachkova, Maksim Shorikov, Vadim Panov, Igor Tyurin |
| 21 | 2017 | Tumor volume measurement optimization in cervical cancer patients with MRI | Maksim Shorikov, Elena Tarachkova, Ilya Gubskiy, Vadim Panov, Nikolay Schimanowsky, Igor Tyurin |
| 22 | 2017 | Imaging spectrum of pediatric adnexal torsion according to the age group | So-Young Yoo, Ji Young Lee, Ji Hye Kim, Tae Yeon Jeon, Se Lin Oh |
| 23 | 2017 | Perineal ultrasonography in grading of female stress urinary incontinence | Doaa Sharaf, Ahmed El Hefnawy, Dia El-din Taha, Mohamed Aboelghar, Bassem Wadie, Tarek Eldiasty |
| 24 | 2017 | Lipiodol Uterine Bathing Effect (LUBE) mechanism of fertility enhancement following HSG — proof of principle in two pilot randomised trials? | Neil Johnson, Shelley Reilly, Lizzi Glanville, Bharati Dhorepatil, Larry Chamley, Andrew Shelling, Cris Print |
| 25 | 2017 | Efficiency of magnetic resonance imaging in prenatal pulmonary hypoplasia and other lung anomalies: comparative study to 3D ultrasound | Noha Behairy, Soha Talaat, Dorria Salem, Maiam Rafaat |
| 26 | 2017 | Endometrial carcinomas in women with polycystic ovarian disease: sonohysterographic findings | Eun Ju Lee |
| 27 | 2017 | Clinical usefulness of endoanal sonographic follow-up in patients with birth injury after sphincteroplasty | Hae Jeong Jeon, Mi Hye Yu, Hee Sun Park, Young Jun Kim, Sung Il Jung, Sang Woo Park, Ung Chae Park |
| 28 | 2017 | MR imaging findings of actinomycosis in the pelvic cavity | Jongchul Kim |
| 29 | 2017 | MRI predictive ability for bladder and parametrial involvement in high risk patients for abnormal placentation: preliminary results | Charis Bourgioti, Konstantina Zafeiropoulou, Stavros Fotopoulos, Konstantinos Chatoupis, Maria Evangelia Nikolaidou, Aristeidis Antoniou, Lia Angela Moulopoulos |
| 30 | 2017 | Validation of prostate imaging reporting and data system version 2 using a MR–ultrasound fusion biopsy in prostate cancer diagnosis | See Hyung Kim |
| 31 | 2017 | MRI-US fusion and additional random biopsy for prebiopsy mp-MRI identified PIRADS 3 Lesion as index lesion — is the mri-us fusion biopsy useful for diagnosis of prostate cancer? | Kye Jin Park, Mi-hyun Kim, Kyoung-sik Cho |
| 32 | 2017 | Different role of PI-RADS category 3 on prebiopsy biparameteric from multiparametric MRI in patients with PSA ≤ 10 ng/mL | Young Joon Lee, Moon Hyung Choi, Seung Eun Jung, Sung Eun Rha, Jae Young Byun |
| 33 | 2017 | Is the multiparametric MRI with Prostate Imaging Reporting and Data system version 2 (PI-RADS v2) sufficiently discovering clinically significant prostate cancer lesions? Per lesion-base, one-to-one radiology-pathology correlation study | Myoung Seok Lee, Min Hoan Moon, Chang Kyu Sung |
| 34 | 2017 | Interobserver agreement in PI-RADS v2 analysis | Myoung Seok Lee, Min Hoan Moon, Chang Kyu Sung |
| 35 | 2017 | Assessment of prostate cancer detection and tumor aggressiveness using diffusion weighted and dynamic contrast-enhanced MR imaging at 3T | Oliwia Kozak, Karolina Markiet, Katarzyna Skrobisz, Anna Szymańska-Dubowik, Michał Studniarek |
| 36 | 2017 | Diffusion Tensor Imaging (DTI) of physiologic urinary bladder in humans: a feasibility study | Isabella Ceravolo, Giovanni Barchetti, Davide Carano, Davide Fierro, Maurizio Del Monte, Valeria Panebianco |
| 37 | 2017 | Comparison of contrast enhancement in bladder tumors of different histopathological grades | Patrik Tufvesson, Malin Helenius, Anders Magnusson |
| 38 | 2017 | Towards a fast, low dose determination of urinary stone types by X-ray dark field radiographic imaging | Tilo Niemann, Iwan Jerjen, Lukas Hefermehl, Zhentian Wang, Rahel A. Kubik-Huch, Marco Stampanoni |
| 39 | 2017 | Fully automatic kidney segmentation and renal cyst detection in CT images | Eyal Klang, Talia Levy, Arnaldo Mayer, Alec Krosser, Yair Rubinstein, Amit Talmon, Neta Gertovsky, Nahum Kiryati, Marianne Michal Amitai, Orith Portnoy |
| 40 | 2017 | Measurement of split renal function before renal donation: a comparison of two different software | Jonas Nyström, Pär Dahlman, Amir Sedigh, Maria Lönnemark, Anders Magnusson, Per Liss |
| 41 | 2017 | Diagnostic yield of CT urography in contemporary practice | Prashant Menon, Sarena Virdee, Sanjay Agarwal |
| 42 | 2017 | Dual energy CT may be a tool in the evaluation of the urinary tract stones chemical composition in everyday practice | Maciej Guziński, Agata Szczurowska, Łukasz Dołowy, Krzysztof Tupikowski, Tomasz Kraśnicki, Marek Sąsiadek |
| 43 | 2017 | Role of dual source dual energy CT in the evaluation of renal masses with quantitative comparison of true and virtual unenhanced images | Abhinaya Shankar, Manavjit Singh Sandhu, Ujjwal Gorsi, Ravi Mohan, Niranjan Khandelwal |
| 44 | 2017 | Accuracy of Iodine quantification using dual source computed tomography — preliminary results with low concentrations | Anders Ahnfelt, Anders Magnusson, Pär Dahlman, Monica Segelsjö, Mats Magnusson |
| 45 | 2017 | Multiparametric non invasive MRI imaging of the kidney | Per Eckerbom, Eleanor Cox, Peter Hansell, Charlotte Buchanan, Jan Weis, Fredrik Palm, Susan Francis, Per Liss |
| 46 | 2017 | Our experiences of setting up a complex pelvic surgical MDT meeting | Cairine Probert, Bethan Hickerton, Robert Macdonald, Paul Rooney, Fraser Smith, Rebecca Wiles |
| 47 | 2017 | Prediction of histologic grade of ureteral urothelial carcinoma on CT urography | Deuk Jae Sung, Sung Tae Hwang, Ki Choon Sim, Na Yeon Han, Beom Jin Park, Min Ju Kim, Sung Bum Cho |
| 48 | 2017 | T1 staging of urinary bladder cancer with an inchworm sign at 3T MRI | Byung Chul Kang, Da Hoon Kim, Hye Ran Hyun |
| 49 | 2017 | Accuracy in the classification of Bosniak IIF, III and IV complex renal cysts in a daily clinical workflow | Janni L.B. Madsen, Stine Degn, Ole Graumann |
| 50 | 2017 | The evaluation of detection rate for urinary calcium stones with low dose CT: Phantom study | Young Taik Oh, Jin Kyem Kim, Sung Yoon Park, Dae Chul Jung |
| 51 | 2017 | Two stage PCNLS: A stage too far? | Rachel Hubbard, Sara Sciacca, Thomas Nash, Gemma Sheehan-Dare, Farhat Kazmi, Nasir Khan |
| 52 | 2017 | Surgical management of urinary stones with abnormal kidney anatomy | Bozkurt OF, Tolga Bozkurt |
| 53 | 2017 | Can CT of the abdomen help to identify the "better kidney" in potential living kidney donors? | Agata Szczurowska, Maciej Guziński, Wojciech Krajewski, Dorota Kamińska, Katarzyna Kościelska-Kasprzak, Antonio Arruza Echevarria, Bartosz Małkiewicz, Paweł Dębiński, Oktawia Mazanowska, Marian Klinger, Marek Sąsiadek |
| 54 | 2017 | A systematic review regarding magnetic resonance imaging's ability to diagnose and subdivide renal tumors among adults compared to histopathology | Janni L. B. Madsen, Louise A. Duus, Lars Lund, Tommy K. Nielsen, Michael Petersen, Ole Graumann |
| 55 | 2017 | The scrotum in the emergency room: ultrasound to the rescue! | Carlos Andre Barroso Oliveira, Gisela Rio, Cristina Silva, Fernanda Gomes, Vasco Mendes |
| 56 | 2018 | Role of positron emission tomography computed tomography in screening metastasis of renal cell carcinoma | A.H. Afifi, Alexandria/EG |
| 57 | 2018 | Stripped fetal vessel sign: MRI appearance of a novel pathological finding in patients with invasive placenta | K. Zafeiropoulou, Athens/GR |
| 58 | 2018 | Epididymal more than testicular abnormalities are associated with the occurrence of antisperm antibodies as evaluated by the MAR test | F. Lotti, Florence/IT |
| 59 | 2018 | When is midline prostatic cysts investigation of clinical value in the work-up of males of infertile couples? | F. Lotti, Florence/IT |
| 60 | 2018 | Performance characteristics of the Prostate Imaging Reporting and Data System (PIRADS) and prostate Magnetic Resonance Imaging (MRI) prior to radical prostatectomy - Which prostate cancers are missed? | J. Wallström, Gothenburg/SE |
| 61 | 2018 | The role of MRI in congenital malformation: Preliminary results of a multidisciplinary approach | L. Russo, Rome/IT |
| 62 | 2018 | Uterine Artery Embolization - Assessment of treatment efficacy in uterine fibroids using pre- and post- procedural MRI, a single centre experience | A.S. Moreira, Maia/PT |
| 63 | 2018 | Microwave Ablation of Renal Tumors | A. Magnusson, Uppsala/SE |
| 64 | 2018 | Implementation of very low dose CT for confirmation of urinary stone presence | D. Raskin, Tel Hashomer/IL |
| 65 | 2018 | What are the Indications for Prophylactic Embolization of Renal Angiomyolipomas, a review of the current evidence in the literature? | T. Geoghegan, Dublin/IE |
| 66 | 2018 | Characterization of Adrenal Lesions at Chemical Shift MRI: Comparison of 1.5T and 3T MRI | I. Hekimsoy, Istanbul/TR |
| 67 | 2018 | Evaluation of adrenal masses using FIESTA (Gradient balanced echo imaging) MRI sequence | G. Pekindil, Istanbul/TR |
| 68 | 2018 | Ureteral metastasis: An uncommon finding of metastatic disease in primary neoplasms. Literature review and our centre's case series report | C. Zwanzger, Barcelona/ES |
| 69 | 2018 | Single system ectopic ureter (SSEU) in girls: A clinical and imaging challenge | S.B. Grover, New Delhi/IN |
| 70 | 2018 | Discrimination of papillary renal cell carcinoma from benign proteinaceous cyst based on iodine and water content on rapid kV-switching dual-energy CT | I. Camlidag, Samsun/TR |
| 71 | 2018 | CT-Guided Cryoablation of Small Renal Masses | L. Aarup Duus, Odense/DK |
| 72 | 2018 | Renal function outcome after ablation or surgical resection of renal tumors - Evaluation using the split renal function | V. Acosta Ruiz, Uppsala/SE |
| 73 | 2018 | Unenhanced attenuation and histogram analysis in CT diagnosis of adrenal tumors | P. Szasz, Olomouc/CZ |
| 74 | 2018 | Proper selection of patients with Stress Urinary Incontinence for physiotherapy: Role of Static and Dynamic MRI | H. Kamal, Cairo/EG |
| 75 | 2018 | The Value of Magnetic Resonance Imaging in the Differential Diagnostic of Ovarian Lesions | B. Olimov, Moscow/RU |
| 76 | 2018 | Comparison of fusion T2-DWI and PET-CT in the detection of lymph node metastasis in patients with cervical cancer | M.A. Serrado, Funchal/PT |
| 77 | 2018 | Imaging spectrum of adnexal pathologies in young girls | S.B. Grover, New Delhi/IN |
| 78 | 2018 | The many faces of Mullerian Duct Anomalies | S.B. Grover, New Delhi/IN |
| 79 | 2018 | Imaging-based prediction of myometrial invasion by invasive placenta with presence of placental bulge sign | L. Poder, San Francisco/US |
| 80 | 2018 | When to perform high resolution imaging for MR evaluation of placenta accreta spectrum? | L. Poder, San Francisco/US |
| 81 | 2018 | Do we need to do 10 biopsies in PIRADS III, IV and V Prostate cancer or are the first 2 targeted biopsies enough? | A. Ikwuagwu, Leeds/UK |
| 82 | 2018 | Evaluation of the impact of orientation differences between multi-parametric MR and ultrasound in biopsy | J. Piper, Cleveland/US |
| 83 | 2018 | Manual adjustment in mpMRI guided prostate biopsy significantly improves the detection rate of prostate cancer: Experience in 400 patients | S. Alessi, Pavia/IT |
| 84 | 2018 | Accuracy of Pre-Operative MRI Staging versus Post-Operative Surgical Histology in Patients who underwent Radical Prostatectomy | J. Chan, Wrexham/UK |
| 85 | 2018 | Prostate Imaging Reporting and Data System (PIRADS) 3 and 4 – 1 year experience from a large UK teaching hospital | K. Fatania, Leeds/UK |
| 86 | 2018 | How to improve the equivocal category PI-RADS score 3? Quantitative multiparametric MRI assessment of prostate cancerous and non-cancerous tissue using correlative histopathology | R. Campa, Rome/IT |
| 87 | 2018 | Is the efficacy of different MR contrast agent important in detection and diagnosis of prostate cancer local recurrence after radical prostatectomy by mpMRI? | F. Kossow, Moscow/RU |
| 88 | 2018 | The Value Of Prostate Magnetic Resonance Imaging To Determine Radial Distance Of Extraprostatic Extension | A. Onay, Ankara/TR |
| 89 | 2018 | Preoperative staging of prostate cancer. Which modality of multiparametric MRI is the most sensitive and specific? | F. Kossow, Moscow/RU |
| 90 | 2018 | Development of an extraprostatic extension imaging score based on multiparametric MRI and histopathology correlations | R. Campa, Rome/IT |
| 91 | 2018 | Characterization of the diffusion tensor imaging properties of normal testicles using high-resolution multi-b value protocol at 3T | N. Nissan, Tel Hashomer/IL |
| 92 | 2018 | MRI of the scrotum for the evaluation of the azoospermic male | K. Skrobisz, Gdansk/PL |
| 93 | 2018 | The added value of scrotal palpation during testicular ultrasound (US): a technical note | J.P. Zawaideh, Genoa/IT |
| 94 | 2018 | Validation of Vesical Imaging Reporting and Data System (VI-RADS) in patients with bladder cancer at a single reference centre | A. Carboni, Rome/IT |
| 95 | 2018 | How Can Dynamic Contrast Enhanced MRI Help in Prediction of Aggressiveness of Urinary Bladder Cancer? | R.T. Abouelkheir, Mansoura/EG |
| 96 | 2018 | Referer confidence in a negative MR of the prostate for the detection of clinically significant prostate cancer | M. Ahmad, Bradford/UK |
| 97 | 2018 | The diagnostic value of MR imaging in predicting muscle invasion of bladder cancer: 3D-FRFSE with oblique-plane versus conventional T2WI three-plane sequence | A. Higashiyama, Osaka/JP |
| 98 | 2019 | DW-MRI predictive factors for radiation-induced vaginal stenosis in patients with cervical cancer | Dr. Martina Sbarra, Dr. Maura Miccò, Dr. Nicola Carlo Bianco, Dr. Maura Campitelli, Prof. Riccardo Manfredi |
| 99 | 2019 | Ovarian cancer subtype and clinical stage in relation to mammographic breast density | Dr. Hanna Sartor, Dr. Maria Bjurberg, Dr. Jenny Brandtstedt, Prof. Karin Jirström |
| 100 | 2019 | The use of radiomics to predict response to treatment in patients with locally advanced cervical cancer | Dr. Luca Russo, Dr. Benedetta Gui, Prof. Gabriella Ferrandina, Dr. Luca Boldrini, Prof. Riccardo Manfredi |
| 101 | 2019 | The role of DWI in the evaluation of pelvic lymph nodes in cervical cancer | Dr. Luca Russo, Dr. Benedetta Gui, Dr. Rosa Autorino, Prof. Gabriella Ferrandina, Prof. Riccardo Manfredi |
| 102 | 2019 | Follow up of patients with low and intermediate-risk prostate cancer by imaging: Can MRI predict upgrading histopathology and withdrawal from active surveillance? | Dr. Sandy Van Nieuwenhove, Dr. Julien Van Damme, Mr. Nicolas Michoux, Prof. Frederic Lecouvet, Prof. Bertrand Tombal |
| 103 | 2019 | Biparametric MRI in men with previous negative or low grade tumor on standard biopsies, MR/US targeted biopsies and prostatectomy: comparison of MRI and whole mount prostatectomy step sections | Ms. Linnea Hansson, Dr. Rafaele Ahlman, Dr. Pontus Röbeck, Dr. Sam Ladjevardi, Dr. Pär Dahlman |
| 104 | 2019 | Donor renal volume and eGFR predicts graft outcome in living donor renal transplants | Dr. David Brennan, Dr. Douglas Mulholland, Dr. Chaudhry Adeel Ebad, Dr. Ruth Dunne, Prof. Martina Morrin |
| 105 | 2019 | Prediction of Clinically Significant Prostate Cancer: Histogram Analysis from Stretched Exponential Model on Diffusion-Weighted Imaging | Dr. Hyun Soo Kim, Prof. Chan Kyo Kim |
| 106 | 2019 | The utility of CT and MRI in detecting male urethral recurrence after radical cystectomy | Dr. Hyun Soo Kim, Prof. Byung Kwan Park |
| 107 | 2019 | Comparison of multiple abbreviated MRI protocols for prostate cancer detection: a multi-reader study in patients referred to radical prostatectomy | Dr. Luisa Di Mico, Dr. Gianluca Giannarini, Dr. Lorenzo Cereser, Prof. Chiara Zuiani, Prof. Rossano Girometti |
| 108 | 2019 | Pitfalls in Prostate Cancer Diagnosis: Avoiding the Unnecessary Biopsies | Dr. Vlad Bura, Dr. Roxana Pintican, Dr. Cristian Popita, Dr. Claudia Moldovanu, Prof. Andrei Lebovici |
| 109 | 2019 | PI-RADS version 2.1 (2019): bound for less unnecessary prostate biopsies | Dr. Vlad Bura, Dr. Roxana Pintican, Dr. Cristian Popita, Dr. Claudia Moldovanu, Prof. Andrei Lebovici |
| 110 | 2019 | Role of age in a MRI-based active surveillance cohort: association with clinical and radiological progression | Mr. Akshay Narayan, Dr. Francesco Giganti, Dr. Vasilis Stavrinides, Prof. Caroline Moore |
| 111 | 2019 | What ADC histogram metric and combination of b-values are appropriate for evaluating prostate cancer on DWI? | Prof. Masamitsu Hatakenaka, Dr. Naomi Koyama, Dr. Koichi Onodera, Dr. Maki Onodera, Dr. Naoya Yama |
| 112 | 2019 | An Audit of the Quality of Renal Tract Opacification in CT Urograms | Dr. James Cusack, Dr. Christopher McLeavy, Dr. Gajraj Sharma |
| 113 | 2019 | Contrast-enhanced ultrasound (CEUS) in the urinary tract | Dr. Prof Shabnam Bhandari Grover, Dr. Hemal Grover, Dr. Mohd Altamash, Dr. Amit Katyan, Prof. Anup Kumar |
| 114 | 2019 | Adrenal washout CT - what's the use | Dr. Khurram Shahzad, Dr. James Lay |
| 115 | 2019 | MDCT evaluation of synchronous primary prostatic carcinoma and other solid malignancies | Prof. Adel El-Badrawy |
| 116 | 2019 | MDCT evaluation of synchronous primary vesical carcinoma and other solid malignancies | Prof. Adel El-Badrawy |
| 117 | 2019 | 1H MR spectroscopy in predicting spermatogenic status of testes with non-obstructive azoospermia | Dr. Alexandra Ntorkou, Prof. Athina Tsili, Prof. Loukas Astrakas, Prof. Nikolaos Sofikitis, Prof. Maria Argyropoulou |
| 118 | 2019 | Malignancy prediction using the IOTA simple rules risk calculation model for definitive characterization of sonographically complex adnexal masses | Dr. Prof Shabnam Bhandari Grover, Dr. Sayantan Patra, Dr. Heena Rajani, Dr. Pratima Mittal, Dr. Geetika Khanna |
| 119 | 2019 | Role of imaging in the evaluation of female infertility: when and how | Dr. Mariana Chaves, Dr. Alexandra Mesquita, Dr. Sofia Amante, Dr. Rafaela Sousa, Dr. David Silva |
| 120 | 2019 | CT findings in post-partum complications: our experience | Dr. Salvatore Persiani, Dr. Benedetta Gui, Dr. Laura Maria Minordi, Dr. Brigida Carducci, Prof. Riccardo Manfredi |
| 121 | 2019 | CT features of postoperative complications after Cytoreductive Surgery and HIPEC in Patients Treated for Peritoneal Malignancies: Our Experience | Dr. Nicola Carlo Bianco, Dr. Maura Miccò, Dr. Benedetta Gui, Dr. Elena Rodolfino, Prof. Riccardo Manfredi |
| 122 | 2019 | Retrospective review of CT imaging features in pathologically confirmed cases of Ovarian torsion | Dr. Patrick Navin, Dr. David Bartlett, Dr. Ashish Khandelwal |
| 123 | 2019 | Establishing, optimising, and adapting a transrectal ultrasound guided prostate biopsy antibiotic protocol in an era of emerging antibiotic resistance | Dr. James Ryan, Ms. Nuala Scanlon, Mr. Kieran O'Malley, Prof. John Murray, Dr. Carmel Cronin |
| 124 | 2019 | Evaluate the effect of varicocele embolization on the Testicular vascularization (TV) assessed by Ultrasensitive Doppler (USD) during Valsalva Manoeuvre (VM) | Prof. Laurence Rocher, Dr. Florian Maxwell, Dr. Cristina Balasa, Dr. Ghina Hindawi, Dr. Jean-Baptiste Debry |
| 125 | 2019 | Renal graft tumours treated with percutaneous ultrasound-guided cryoablation | Dr. Janni Lynggård Bo Madsen, Dr. Tommy K. Nielsen, Dr. Jan Solvig, Dr. Ole Graumann |
| 126 | 2019 | Estimation of Renal Function using Kidney Perfusion CT | Prof. Sung Bin Park |
| 127 | 2019 | Theatre based Renal access for PCNL: Is the higher dose justified? | Dr. Rachel Hubbard, Ms. Isabel Barraclough, Dr. Ajay Singaravelou, Dr. Farhat Kazmi, Dr. Nasir Khan |
| 128 | 2019 | Trans arterial embolization for Emergency Genitourinary Interventional patients | Prof. DAE HYUN Hwang |
| 129 | 2019 | Useful fluoroscopic and sonographic procedures in Urogenital Radiology – a manual for trainee Radiologists | Dr. David Ryan, Dr. Amy O'Brien, Dr. Adam Roche, Dr. Roisin MacDermott, Dr. Ronan Ryan |
| 130 | 2021 | A multi-variate correlation analysis for determining the clinical and pathology features associated with VI-RADS assess | Martina Pecoraro, Marco Bicchetti, Sara Lucciola, Emanuele Messina, Ailin Dehghanpour, Martina Pisciotti, Valeria Panebianco |
| 131 | 2021 | Primary Detection of Prostate Cancer: MRI In-Bore vs MRI-Transrectal Ultrasound Fusion Targeted Techniques | Emanuele Messina, Martina Pecoraro, Cipollari Stefano, Marco Bicchetti, Maurizio Del Monte Martina Lucia Pisciotti, Sara Lucciola, Ailin Dehghanpour, Valeria Panebianco |
| 132 | 2021 | Systematic review of the incidence of adverse reactions after intra-arterial ioversol administration | Ilona A. Dekkers, Aart J. van der Molen, Ibrahim Bedioune, Elisabeth Darmon-Kern |
| 133 | 2021 | Systematic Review of the Incidence of Hypersensitivity Reactions and Post-Contrast Acute Kidney Injury after IV Administration of loversol in more than 57,000 Patients | Aart J. van der Molen, Ilona A. Dekkers, Ibrahim Bedioune, Elisabeth Darmon-Kern |
| 134 | 2021 | Volumetric apparent diffusion coefficient histogram analysis of the testes in nonobstructive azoospermia | Athina C. Tsili, Nikolaos Sofikitis, Loukas Astrakas, Anna Goussia, Thomas, Benekos, Aris Kaltsas, Vasileios P. Maliakas, Maria I. Argyropoulou |
| 135 | 2021 | A practical approach to borderline ovarian tumours: tips and pearls | Ana Sofia Alves, Ana Félix, Teresa Margarida Cunha |
| 136 | 2021 | Scrotal tumors: ultrasonography (US) role in urgency diagnosis and therapeutic decision | Carolina Zaghini, Felipe Figueroa, Giovana Helfenstein, Ricardo Azze Natel, Victor Jabour, Antonio Rahal Junior, Miguel Francisco Neto, Marcos Queiroz |
| 137 | 2021 | Use of hemostatic matrices in renal biopsy pathways: why and why not? | Ricardo Natel, Beatriz Ahmad, Bruno Pasquini, Carolina Zaghini, Priscila Falsarella, Antonio Rahal, Fabio Vieira, Rodrigo Gobbo |
| 138 | 2021 | Percutaneous tagging guided by computer tomography (CT) of positive Ga-prostate specific membrane antigen (PSMA) pelvic | Carolina Zaghini, Giovana Helfenstein, Ricardo Azze-Natel, Ana Brocoletti, Victor Jabour, Antonio Rahal-Jr, Paulo Silverio, Marcos Queiroz |
| 139 | 2021 | Penile neoplasia: aspects in different methods of imaging exams and emerging modalities in the diagnostic arsenal | Carolina Zaghini, Ana Beatriz Brocoletti, Giovana Helfenstein, Ricardo Azze Natel, Antonio Rahal Junior, Bruno Schmid, Felipe Nasser, Rodrigo Gobbo Garcia |
| 140 | 2021 | Risk factors to perform percutaneous kidney biopsy (PKB): knowing and preventing | Ricardo Natel, Ana Brocoletti, Giovana Helfenstein, Carolina Zaghini, Victor Jabour, Priscila Falsarella, Antonio Rahal, Rodrigo Gobbo |
| 141 | 2021 | Comparison between percutaneous cryoablation (PRCA) and partial nephrectomy (PN) | Carolina Zaghini, Ana Beatriz Brocoletti, Felipe Figueroa, Ricardo Azze Natel, Rodrigo Gobbo Garcia |
| 142 | 2021 | ultrasound guided by multiparametric magnetic resonance with target biopsies (mpMRI-TRUS) x 14 prostatic biopsy fragment | Beatriz Ahmad, Carolina Zaghini, Bruno Pasquini, Ricardo Azze-natel, Guilherme Mariotti, Antonio Rahal-Jr, Gustavo Lemos, Rodrigo Gobbo-Garcia |
| 143 | 2021 | Urethral cancer: aspects in different methods of imaging exams | Ricardo Natel, Beatriz Ahmad, Bruno Pasquini, Carolina Zaghini, Victor Jabour, Priscila Falsarella, Antonio Rahal, Rodrigo Gobbo |
| 144 | 2022 | Artificial intelligence to predict the surgical outcome in ovarian cancer using preoperative CT: preliminary result | Alessio Perazzolo, Giacomo Avesani, Lorenzo Elia, Alessia Cipriani, Camilla Panico, Benedetta Gui, Riccardo Manfredi |
| 145 | 2022 | Pulmonary and peritoneal metastases in patients with uterine leiomyosarcoma: assessing tumour volume doubling time | Riccardo Ciabattoni, Ana Teresa Vilares, Raquel Baptista Dias, Teresa Margarida Cunha |
| 146 | 2022 | Radiological and clinical progression of prostate cancer: update on a UK cohort utilising the PRECISE recommendations | Cameron Englman, Vasilis Stavrinides, Aiman Haider, Alex Freeman, Alex Kirkham, Mark Emberton, Clare Allen, Caroline Moore, Francesco Giganti |
| 147 | 2022 | Diagnostic Accuracy and Observer Agreement of the MRI Prostate Imaging for Recurrence Reporting Assessment Score | Martina Pecoraro, Ludovica Laschena, Baris Turkbey, Andrei Purysko, Rossano Girometti, Valeria Panebianco |
| 148 | 2022 | Improving prostate cancer detection on MRI using PSAD: additional benefit from zone-specific analyses? | Charlie Hamm, Konrad Froböse, Lukas Baumgärtner, Tobias Penzkofer |
| 149 | 2022 | Predictive MDCT Models for Characterization of Renal Carcinoma and Oncocytoma: Three Phases Nomogram Analytical Approach | Haytham Shebel, Doaa Sharaf, Hebba Abou El Atta, Tarek El-Diasty |
| 150 | 2022 | Is there a learning curve when performing percutaneous renal tumor ablation? | Anders Magnusson, Anton Sundelius, Vanessa Acosta Ruiz, Mats Ahlberg, Einar Brekkan, Pär Dahlman |
| 151 | 2022 | Decreased renal perfusion during acute kidney injury in COVID-19 patients evaluated by magnetic resonance imaging | Tomas Luther, Per Eckerbom, Eleanor Cox, Miklós Lipcsey, Michael Hultström, Jan Weis, Susan Francis, Robert Frithiof, Per Liss |
| 152 | 2022 | 3.0 T Diffusion Tensor Imaging and Tractography of the testis in nonobstructive azoospermia: an observational study | Ourania Pappa, Christina Bougia, Vasileios Maliakas, Aris Kaltsas, Nikolaos Sofikitis, Maria Argyropoulou, Athina Tsili |
| 153 | 2022 | Assessment of early response to neoadjuvant chemotherapy in multi-site HGS ovarian cancer using hyperpolarized-13C-MRI | Vlad Bura, Lucian Beer, Ramona Woitek, Mary McLean, Cara Brodie, Mercedes Jimenez-Linan, Ferdia Gallagher, Kevin Brindle, Evis Sala |
| 154 | 2022 | What Can Radiogenomics Offer to the Uroradiologist? – Results from a Systematic Review | Kristine Bærentz Holst, Anne Rix Arildskov, Frederik Jager Bruun, Lotte Harries Hasselbalch, Sine Hvid Rasmussen, Consuelo Borrás, Rebeca Mirón Mombiela |
| 155 | 2022 | Prediction of surgical outcomes of interval debulking surgery in advanced ovarian cancer after neoadjuvant chemotherapy | Na Young Kim, Dae Chul Jung, Kyung Hwa Han, Jung-Yun Lee |
| 156 | 2022 | Differentiating renal epithelioid angiomyolipoma from clear cell carcinoma: using a radiomics model with CT findings | Taek Min Kim, Hyungwoo Ahn, Hyo Jeong Lee, Jeong Yeon Cho, Sung Il Hwang, Sang Youn Kim |
| 157 | 2022 | Renal function 6 months after robot-assisted partial nephrectomy and percutaneous cryoablation of renal cell carcinoma | Louise Aarup Duus, Theresa Junker, Benjamin Schnack Brandt Rasmussen, Louise Aarup Duus, Michael Pedersen, Lars Lund, Ole Graumann |
| 158 | 2022 | Is isotope renography still a routine in live kidney donor side selection in the era of computed tomography volumetry? | Bedier Ali-El-Dein, Ahmed Galal, Huda Refaie, Mohamed Mashaly, Ayman Refaie, Yaser Osman |
| 159 | 2022 | Does the kidney really swell during an acute urinary tract obstruction? | Klara Sahlén, Olle Lönnemark, Anders Magnusson |
| 160 | 2022 | CT Cryotherapy for the Small Renal Mass: Real Life Outcomes from the North West of England | Maame Akua Akobeng, Nishant Omar, Jonathan Tuck, Hazel Warburton, Will Brady |
| 161 | 2022 | Cryoablation treatment of renal tumors located close to the pelvoureteric junction, proximal ureter or collecting system | Ludvig Tivell, Mats Ahlberg, Einar Brekkan, Pär Dahlman, Maria Lönnemark, Anders Magnusson |
| 162 | 2022 | Long term follow-up after ct guided ablation of renal cell carcinomas: oncological outcomes | Anton Sundelius, Vanessa Acosta Ruiz, Mats Ahlberg, Einar Brekkan, Pär Dahlman, Maria Lönnemark, Anders Magnusson |
| 163 | 2022 | A randomised controlled trial on the role of MRI for prostate cancer screening (PROSA): Preliminary results | Emanuele Messina, Martina Pecoraro, Marco Bicchetti, Martina Lucia Pisciotti, Carlo Catalano, Valeria Panebianco |
| 164 | 2022 | Which biopsy method is superior for the diagnosis of prostate cancer? A prospective single-center study from Greece | Georgios Agrotis, Katerina Vassiou, Athanasios Oikonomou, Iannis Tsougos, Vasileios Tzortzis, Marianna Vylchou |
| 165 | 2022 | Impact of second reads of equivocal lesions in the one-stop prostate MRI clinic | Alexandre Woernle, Louise Dickinson, Douglas Pendse, Daniel Heffernan Ho, Navin Ramachandran, John Hines, Caroline Moore, Clare Allen, Francesco Giganti |
| 166 | 2022 | Artificial intelligence (Quantib®Prostate) to improve cancer detection in transperineal MRI/ultrasound fusion biopsies | Günzel Karsten, Schlegel Jakob, Heinrich Stefan, Busch Jonas, Hinz Stefan, Magheli Ahmed |
| 167 | 2022 | Predictive role of Node-RADS in patients with PCa candidates to radical prostatectomy and extended lymph node dissection | Ludovica Laschena, Sara Lucciola, Martina Lucia Pisciotti, Emanuele Messina, Carlo Catalano, Valeria Panebianco |
| 168 | 2022 | MRI assessment of prostate cancer recurrence using the PI-RR system: diagnostic accuracy and interobserver reliability | Paolo Niccolò Franco, Sofia Frade Dos Santos, Alejandra García Baizán, Sandro Sironi, María Milagros Otero García |
| 169 | 2022 | The learning curve in bladder MRI using VI-RADS assessment score during an interactive dedicated training program | Ailin Dehghanpour, Martina Pecoraro, Antonella Borrelli, Marco Bicchetti, Carlo Catalano, Valeria Panebianco |
| 170 | 2022 | Does the VI RADS score help machine learning show that bladder cancer has invaded the muscles? | Merve Şam Özdemir, Sena Azamat, Harun Özdemir |
| 171 | 2022 | Efficacy and safety of gadopiclenol for MRI of body regions: Results from the Phase III PROMISE trial | Orlin Zlatarski, Galina Kirova-Nedyalkova |
| 172 | 2022 | A review of international pharmacovigilance databases to assess symptoms associated with gadolinium exposure | Eric Lancelot, Alvin Joseph, Imran Shahid |
| 173 | 2022 | How much can we reduce the amount of excreted iodine in waste water by collecting the first urine after CT | Helena Dekker, Luuk Oostveen, Willem Jan Van der Woude, Mathias Prokop |
| 174 | 2022 | Testicular torsion without testicular pain: how frequent in adults? | Gugliemo Mantica, Jeries Paolo Zawaideh, Michele Bertolotto, Carlo Terrone, Lorenzo Derchi |
| 175 | 2022 | The European Academy of Andrology (EAA) ultrasound study on healthy, fertile men: scrotal ultrasound reference ranges | Francesco Lotti, EAA US Network |
| 176 | 2022 | Diffusion properties of the normal epididymis on a 3.0 T | Christina Bougia, Maria Argyropoulou, Athina C. Tsili |
| 177 | 2022 | Application of AI Software on CT images for the evaluation of Sarcopenia in Patients with Genitourinary Tumors | Antonella Borrelli, Martina Pecoraro, Emanuele Messina, Ailin Dehghanpour, Sara Lucciola, Valeria Panebianco |
| 178 | 2022 | Role of O-RADS MRI to classificate indeterminate ovarian masses. Difference between senior and junior radiologist | Lledo Cabedo, Carmen Sebastia, Pere Fuste, Meritxell Munmany, Isabel Matas, Blanca Paño, Carlos Nicolau |
| 179 | 2022 | Value of MRI in parametrial invasion assessment and association with prognosis in locally advanced cervical cancer | Luca D'Erme, Luca Russo, Michela Lupinelli, Benedetta Gui, Maura Miccò, Salvatore Persiani, Matteo Mangialardi, Maria Gabriella Ferrandina, Riccardo Manfredi |
| 180 | 2022 | The role of simultaneous 18F-FDG PET/MRI in the assessment of primary cervical cancer – a pilot study | Elisabeth Hedlund, Björg Jónsdóttir, Håkan Ahlström, Karin Stålberg |
| 181 | 2022 | Cervical Cancer in Cape Verde: insights into the reality of a Sub-Saharan African country | Ana Teresa Vilares, Riccardo Ciabattoni, Teresa Margarida Cunha |
| 182 | 2022 | Secrets of uterine junctional zone | Härmä Kirsi, Binda Aleksandra, Heverhagen Johannes |
| 183 | 2022 | T2*-weighted imaging performance in the detection of deep endometriosis: comparison with conventional MRI sequences | Paolo Niccolò Franco, Simona Annibali, Sara Viganò, Caterina Cazzella, Pietro Andrea Bonaffini, Paolo Marra, María Milagros Otero García, Caroline Reinhold, Sandro Sironi |

Abbreviation: ESUR, European Society of Urogenital Radiology.

**Table S2. Publication outcomes by congress year**

| **Year** | **Total oral presentations** | **Pre-congress** | **Ambiguous congress-month** | **Eligible** | **Published during full follow-up** | **Publication proportion** |
| --- | --- | --- | --- | --- | --- | --- |
| 2017 | 55 | 7 | 0 | 48 | 19 | 39.6% |
| 2018 | 42 | 3 | 0 | 39 | 21 | 53.8% |
| 2019 | 32 | 2 | 0 | 30 | 15 | 50.0% |
| 2021 | 14 | 1 | 0 | 13 | 5 | 38.5% |
| 2022 | 40 | 9 | 2 | 31 | 19 | 61.3% |
| **Total** | **183** | **22** | **2** | **161** | **79** | **49.1%** |

**Table S3. Publication outcomes by urogenital subspecialty**

| **Subspecialty** | **Eligible** | **Published during full follow-up** | **Publication proportion** |
| --- | --- | --- | --- |
| Prostate | 43 | 17 | 39.5% |
| Upper urinary tract | 42 | 24 | 57.1% |
| Female reproductive | 33 | 18 | 54.5% |
| Male reproductive | 20 | 15 | 75.0% |
| Lower urinary tract | 16 | 4 | 25.0% |
| Other | 7 | 1 | 14.3% |
| **Total** | **161** | **79** | **49.1%** |

These subgroup analyses were exploratory and should be interpreted descriptively because several categories contained small numbers of presentations.

**Table S4. Publication outcomes by imaging modality**

| **Modality** | **Eligible** | **Published during full follow-up** | **Publication proportion** |
| --- | --- | --- | --- |
| MRI | 68 | 39 | 57.4% |
| CT | 35 | 19 | 54.3% |
| Unspecified / non-modality-specific | 24 | 3 | 12.5% |
| Ultrasound | 18 | 8 | 44.4% |
| Multimodality | 8 | 5 | 62.5% |
| Nuclear medicine | 2 | 1 | 50.0% |
| Other | 2 | 2 | 100.0% |
| PET | 2 | 0 | 0.0% |
| Radiography | 2 | 2 | 100.0% |
| **Total** | **161** | **79** | **49.1%** |

These subgroup analyses were exploratory and should be interpreted descriptively because several categories contained small numbers of presentations.

**Table S5. Publication outcomes by country group**

| **Country group** | **Eligible** | **Published during full follow-up** | **Publication proportion** |
| --- | --- | --- | --- |
| Italy | 27 | 20 | 74.1% |
| South Korea | 14 | 7 | 50.0% |
| Sweden | 14 | 6 | 42.9% |
| United Kingdom | 17 | 4 | 23.5% |
| Other countries | 89 | 42 | 47.2% |
| **Total** | **161** | **79** | **49.1%** |

These subgroup analyses were exploratory and should be interpreted descriptively because several categories contained small numbers of presentations.

**Table S6. AI-related versus non-AI presentations**

| **Category** | **Eligible** | **Published during full follow-up** | **Publication proportion** |
| --- | --- | --- | --- |
| AI-related | 9 | 7 | 77.8% |
| Non-AI | 152 | 72 | 47.4% |
| **Total** | **161** | **79** | **49.1%** |

Fisher’s exact test: p = 0.095. Because only nine AI-related presentations were eligible after exclusion of pre-congress publications, this subgroup should be interpreted as descriptive and hypothesis-generating.
